# The N-Terminal of Aquareovirus NS80 Is Required for Interacting with Viral Proteins and Viral Replication

**DOI:** 10.1371/journal.pone.0148550

**Published:** 2016-02-12

**Authors:** Jie Zhang, Hong Guo, Qingxiu Chen, Fuxian Zhang, Qin Fang

**Affiliations:** 1 State Key Laboratory of Virology, Wuhan Institute of Virology, Chinese Academy of Sciences, Wuhan, 430071, China; 2 University of Chinese Academy of Sciences, Beijing, China; University of Pittsburgh School of Medicine, UNITED STATES

## Abstract

Reovirus replication and assembly occurs within viral inclusion bodies that formed in specific intracellular compartments of cytoplasm in infected cells. Previous study indicated that aquareovirus NS80 is able to form inclusion bodies, and also can retain viral proteins within its inclusions. To better understand how NS80 performed in viral replication and assembly, the functional regions of NS80 associated with other viral proteins in aquareovirus replication were investigated in this study. Deletion mutational analysis and rotavirus NSP5-based protein association platform were used to detect association regions. Immunofluorescence images indicated that different N-terminal regions of NS80 could associate with viral proteins VP1, VP4, VP6 and NS38. Further co-immunoprecipitation analysis confirmed the interaction between VP1, VP4, VP6 or NS38 with different regions covering the N-terminal amino acid (aa, 1–471) of NS80, respectively. Moreover, removal of NS80 N-terminal sequences required for interaction with proteins VP1, VP4, VP6 or NS38 not only prevented the capacity of NS80 to support viral replication in NS80 shRNA-based replication complementation assays, but also inhibited the expression of aquareovirus proteins, suggesting that N-terminal regions of NS80 are necessary for viral replication. These results provided a foundational basis for further understanding the role of NS80 in viral replication and assembly during aquareovirus infection.

## Introduction

Aquareoviruses, the isolates from aquatic animals, are members of the genus *Aquareovirus* in the family *Reoviridae* [1]. Grass carp reovirus (GCRV) has been recognized as the most pathogenic among the isolated aquareoviruses [2]. The particle of GCRV is non-enveloped with icosahedral symmetry enclosing a segmented double-stranded RNA genome in its central core. The eleven genomic segments encode seven structural proteins (VP1 to VP7) and five nonstructural proteins (NS80, NS38, NS31, NS26 and NS16) [3, 4]. Like other reoviruses, the outer-capsid proteins VP5 and VP7 are required for viral entry into host cells during infection, whilst other proteins VP1-VP4 and VP6 compose the inner core of aquareovirus, which play an important role in viral replication [5–8].

Similar to other viruses, the replication and assembly of reoviruses take place in specific intracellular compartments called viral inclusion bodies (VIBs), viral factories (VFs) or virioplasms [9–12]. Previous studies have demonstrated that the nonstructural protein μNS of mammalian orthoreoviruses (MRV) and avian orthoreoviruses (ARV) formed inclusion bodies when expressed alone in cells or during viral infection [9, 13–16]. And also μNS could retain the nonstructural protein σNS and inner-capsid proteins within viral factories by interacting with these proteins [9, 14, 17–20]. Moreover, host ribosomal subunits and related proteins involved in translation were found to colocalize with inclusion bodies in MRV [11]. Besides, nonstructural protein NSP5 of rotavirus was able to form virioplasms when expressed alone in cells [21, 22]. Further investigation indicated that rotavirus inner-capsid proteins VP1, VP2, VP3 and VP6 could be also recruited into its inclusions by interacting with NSP5 or NSP2 [23–27]. In addition to the viral proteins, newly synthesized viral RNAs were also located within viral inclusion bodies [18, 28–30].

The nonstructural protein NS80 of aquareovirus, encoded by genome segment S4, is consisted of 742 amino acids (aa) with a molecular weight of about 80 kDa [31]. Previous study in our lab has demonstrated that NS80 can form viral inclusion bodies in singly expressed or infected cells, and these virioplasms have no colocalization with poly-ubiquitin in infected and transfected cells, indicating that NS80-derived inclusion bodies in cells are not induced by misfolding proteins. And the C-terminal regions including His569 and Cys571 in the intercoil region of NS80 were identified to be crucial for viral inclusions formation [32]. In addition, NS80 is also found to associate with aquareovirus inner-capsid proteins (VP1-VP4, VP6), the putative single-stranded RNA (ssRNA) binding protein NS38 and newly synthesized viral RNAs in both transfected and infected cells [32, 33]. More recently, a report indicated that NS80 was able to coordinate the expression of viral structural proteins and viral replication [33]. To further understand the role of NS80 played in viral replication and assembly, it is necessary to identify functional regions of NS80 that interacted with viral proteins during infection.

In this present study, the functional regions of NS80 associated with proteins VP1, VP4, VP6 and NS38 was defined using RV NSP5-based protein association platform by immunofluorescence assays. And the interaction regions between NS80 and VP1, VP4, VP6, or NS38 were confirmed by co-immunoprecipitation analysis. It was found that the N-terminal (aa 1–471) of NS80 was required for interacting with VP1, VP4, VP6 and NS38. Notably, the N-terminal of NS80 was found to play an important role in supporting viral replication in NS80 shRNA-based replication complementation assays. These results will expand our knowledge to understand the role of NS80 in aquareovirus replication.

## Material and Methods

### Cells, Antibodies and Reagents

HEK 293T cells and Vero cells were grown in Dulbecco’s modified Eagle medium (DMEM) (Gibco-BRL) supplemented with 10% fetal bovine serum (FBS) and 100 U/ml of penicillin and streptomycin. CIK (*Ctenopharyngodon idellus* kidney) cells were grown in minimum essential medium (MEM) (Gibco-BRL) supplemented with 10% FBS respectively. GCRV-873 isolated and stored in the author’s laboratory was propagated in CIK cells as described previously [34].

GCRV VP1, VP4, VP6, VP7, NS38 and NS80 antibodies were generated and stored in our laboratory [31, 35–38]. Mouse anti-Flag monoclonal antibody (mAb) was purchased from Abmart (Shanghai, China). Mouse monoclonal IgG2b isotype control antibody was purchased from eBioscience Inc. (San Diego, CA). Mouse anti-β-actin mAb, rabbit anti-poly-ubiquitin and anti-vimentin polyclonal antibodies (pAbs) were purchased from Proteintech (Wuhan, China). Alexa Fluor^®^ 488 or 568 donkey anti-rabbit IgG (H+L) antibody, Alexa Fluor^®^ 488 or 568 donkey anti-mouse IgG (H+L) antibody and Lipofectamine 2000 (Lipo2000) were purchased from Invitrogen Co. (Invitrogen, Carlsbad, USA).

### Plasmids Construction

All enzymes, except for T4 DNA ligase (New England BioLabs, Massachusetts), used for cloning procedures were purchased from Takara (Dalian, China). Plasmids pCI-neo-VP1, pCI-neo-VP4, pCI-neo-VP6, pCI-neo-NS38, pCI-neo-NS80, pCI-neo-NS80(130–742), pCI-neo-NS80(268–742), pCI-neo-NS80(335–742), pCI-neo-NS80(471–742), and pCI-neo-NS80(485–742) were prepared previously and stored in our laboratory [32, 33, 35–37]. pcDNA3-NSP5-GFP plasmid was obtained from Dr. Francesca Arnoldi. To make pGFP-NSP5 plasmid, PCR was performed by using pcDNA3-NSP5-GFP as a template, and was cloned into pGFP vector. To construct a series of NS80 N-terminal fragment fusion plasmids (S1 Table), PCR was performed by using pCI-neo-NS80 as a template. At first, these amplified NS80 fragments were cloned into the *BamH*I and *EcoR*I sites of the pGFP-N3, and then these plasmids were digested with *Nde*I and *BsrG*I and ligated into *Nde*I and *BsrG*I-digested GFP-NSP5 to generate NS80 fragment fusion plasmids. To build the plasmids Flag-NS80(1–130), Flag-NS80(1–268), Flag-NS80(56–268), and Flag-NS80(56–471), PCR was performed by using pCI-neo-NS80 as a template, these amplified NS80 fragments were digested with *BamH*I and *EcoR*I and ligated into *BamH*I and *EcoR*I-digested pCI-neo or pCMV-Flag vector. The method to construct p-NS38-GFP-NSP5 was as described above. To construct plasmid pCI-neo-NS80(56–742), PCR was performed by using pCI-neo-NS80 as a template, the amplified DNA fragments were digested with *EcoR*I and *Xba*I and ligated into *EcoR*I and *Xba*I-digested pCI-neo vector. To make plasmid pCI-neo-NS80_886m_ (which three silent mutations were introduced into the NS80 shRNA_886_ sequence), PCR was performed by using pCI-neo-NS80 as a template, the following mutagenic primers were used to produce of pCI-neo-NS80_886m_, the forward primer was 5’-CCGATGTACTGCACCGGCAAAGAACGTCACTTCGAAC-3’ and the reverse primer was 5’-GTTCGAAGTGACGTTCTTTGCCGGTGCAGTACATCGG-3’. For each primer, the nucleotide change to give the desired amino acid substitution is underlined. All recombinant plasmids were confirmed by sequencing. Primer sequences used in PCRs are listed in S2 Table.

### Immunofluorescence Assay

Immunofluorescence assays were performed as previously described [32]. Briefly, Vero cells were transfected with indicated plasmids according to the user manual of Lipofectamine 2000 and fixed at 24 h post-transfection, and then fixed in 4% paraformaldehyde. After being permeabilized by Triton X-100, all cells were incubated with the appropriate Alexa Fluor-labeled secondary antibodies following incubation with primary antibodies diluted in blocking buffer. Alexa Fluor^®^ 488 or 568 donkey anti-rabbit IgG (H+L) antibody and Alexa Fluor^®^ 488 or 568 donkey anti-mouse IgG (H+L) antibody were used in 1/400 dilution. After each incubation step, cells were washed extensively with PBS. DAPI staining was applied to detect the cell nucleus. All samples were observed using Olympus-IX51 inverted microscope.

### Co-Immunoprecipitation Assay and Western Blot Analysis

Co-immunoprecipitation (co-IP) assays were performed as previously described [39]. Briefly, HEK 293T cells were co-transfected with 10 μg of each indicated expression plasmids. Transfected cells were harvested at 36 h post-transfection and lysed on ice with 700 μl of lysis buffer. For each immunoprecipitation, a 0.5 ml aliquot of lysate was incubated with 0.5 μg of the anti-Flag mAb or nonspecific mAb (IgG2b isotype matched with anti-Flag mAb) and 30 μl of a 1:1 slurry of Protein A/G Plus-agarose (Santa Cruz, California) for at least 4 h or overnight at 4°C. The beads were washed four times with 1 ml of lysis buffer containing 500 mM NaCl and then subjected to Western blot analysis. All co-IP assays were repeated three times, similar data were obtained, and a typical blot was shown.

Western blot analysis was performed as previously described [40]. Briefly, whole-cell extracts were subjected to 10% SDS-PAGE and transferred to PVDF membranes, followed by blocking with 5% nonfat milk in Tris-buffered saline-Tween (TBST) and probed with the indicated primary antibodies at 37°C for 2 h. After washing with TBST, the membrane was incubated with alkaline phosphatase (AP)-conjugated goat anti-rabbit IgG or goat anti-mouse IgG. Specific protein bands were developed by 5-bromo-4-chloro-3-indolylphosphate (BCIP)-nitroblue tetrazolium (NBT).

### RNA Interference Experiments

The primers of shRNA-control and NS80 shRNA_886_ (which named shRNA-2 in Yan *et*.*al* study) targeted NS80 nucleotide sequence from 886 to 906 were listed in Table 1 and cloned into pcDNA^™^6.2-GW/EmGFP-miR vectors (Invitrogen, Carlsbad, USA). RNA interference experiments were performed as previously described [33]. Briefly, CIK cells were co-transfected with shRNA-control or NS80 shRNA_886_ and either with pCI-neo vector, pCI-neo-NS80_886m_, or pCI-neo-NS80(471–742) plasmid, and then infected with GCRV at MOI of 1 at 24 h post-transfection. Following incubation at 28°C for 30 min, the inoculum was removed and rinsed three times with MEM, subsequently incubated in 2% MEM. Cell supernatant was harvested at 24 h post-infection and viral titers were determined by plaque assays [41].

**Table 1:** Oligonucleotides used to produce shRNA expression vectors.

construct	sequences of shRNA (5,-3,)
shRNA-control	F: TGCTGAAATGTACTGCGCGTGGAGACGTTTTGGCCACTGACTGACGTCTCCACGCAGTACATTT
	R:CCTGAAATGTACTGCGTGGAGACGTCAGTCAGTGGCCAAAACGTCTCCACGCGCAGTACATTTC
NS80 shRNA_886_	F: TGCTGTTCGAAGTGACGCTCCTTACCGTTTTGGCCACTGACTGACGGTAAGGAGTCACTTCGAA
	R: CCTGTTCGAAGTGACTCCTTACCGTCAGTCAGTGGCCAAAACGGTAAGGAGCGTCACTTCGAAC

Letters in bold show sequence corresponding to NS80 genome. And NS80 shRNA_886_ targets NS80 nucleotide sequence from 886 to 906.

## Results

### Construction of NSP5-Based Protein Association Platform

**Fig 1:**
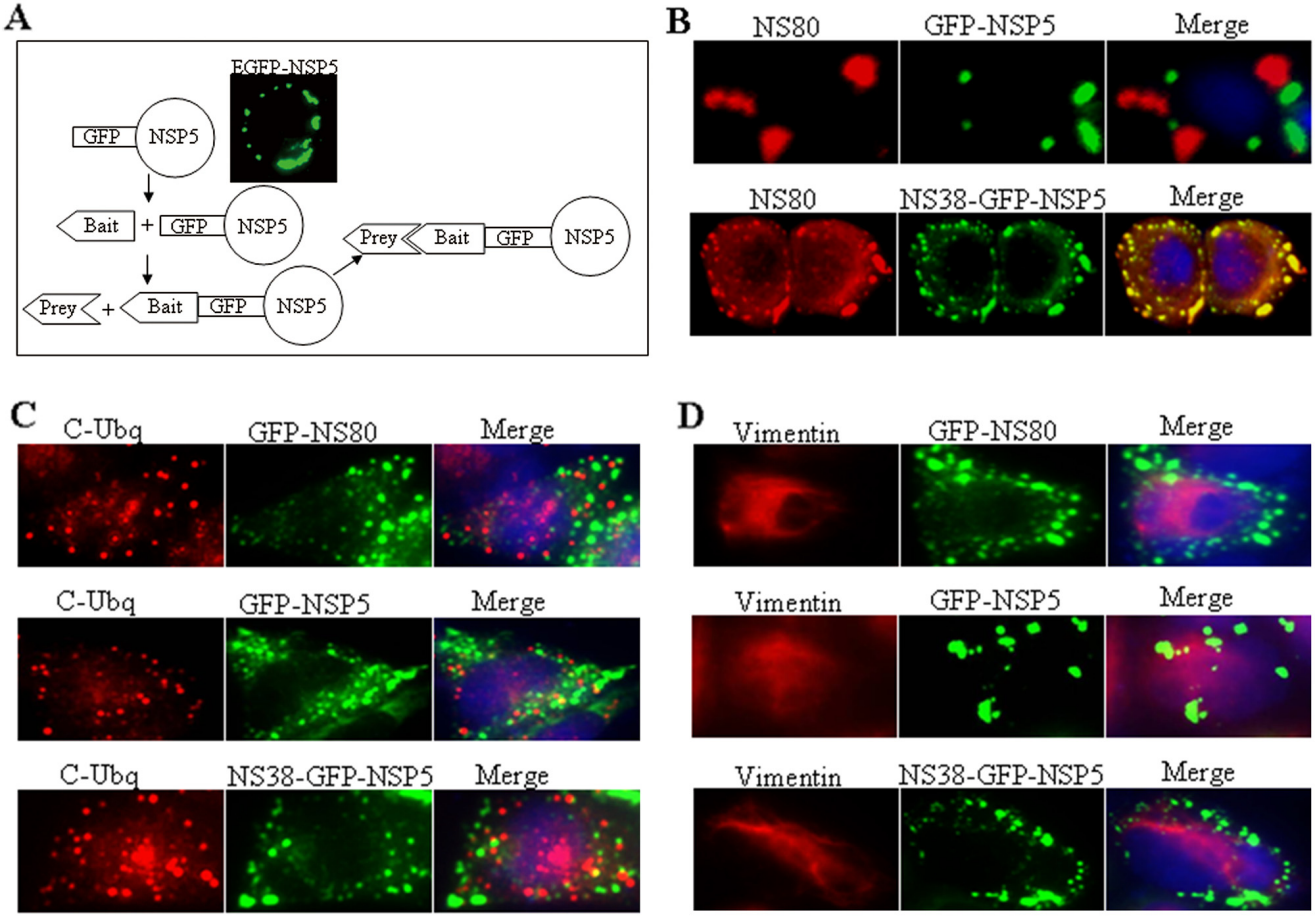
Construction of NSP5-based protein association platform. (A) Principle of the NSP5-based protein association platform. Plasmids expressing NS80 fragments (bait protein) fused to GFP-NSP5 and viral proteins (prey protein) are co-transfected into Vero cells. If prey protein associate with bait protein, it will be recruited into viral inclusion bodies. (B) NS80 and GFP-fused NSP5 form non-overlapping structures. Vero cells co-transfected with pCI-neo-NS80 and either GFP-NSP5 or NS38-GFP-NSP5 were fixed at 24 h post-transfection. Viral inclusion bodies were visualized by staining with NS80 pAbs followed by Alexa 568-conjugated donkey anti-rabbit IgG (red). Cell nuclei (blue) were stained with DAPI. (C and D) Vero cells were transfected with GFP-NS80, GFP-NSP5, or NS38-GFP-NSP5. At 24 h post-transfection, cells were fixed and stained with poly-ubiquitin or vimentin pAbs, and then followed by Alexa Fluor^®^ 568-conjugated donkey anti-rabbit IgG (red). Cell nuclei (blue) were stained with DAPI. The images were obtained by fluorescence microscopy using a 40 × objective.

It is known that NS80 is able to form viral inclusion bodies in both transfected and infected cells, and the C-terminal is responsible for inclusions formation [32]. It was also identified that full-length NS80 bear interactions with inner-capsid proteins VP1, VP4, VP6 and nonstructural protein NS38 during viral infection [32, 33]. However, it is difficult to identify association sequence of NS80 with these known proteins only using NS80 self-formed inclusions phonotype. Previous study reported that the single rotavirus NSP5 is able to form viral factories in transfected cells, which can be used as a fusion protein to identify protein-protein interactions (32). To determine whether exogenous NSP5 could be used as a fusion protein to detect associations of aquareovirus proteins, NSP5-based platform was generated to map regions of aquareovirus NS80 required for associating with other viral proteins. The strategy for constructing the NSP5-based aquareovirus protein-protein association platform was outlined in Fig 1A. Based on the association elements, the capacity of the association platform was confirmed by a known interaction between NS80 (as a prey protein) and NS38 that fused to GFP-NSP5 (as a bait protein). The inherent fluorescence of GFP was used to detect GFP-NSP5 or NS38-GFP-NSP5. When NS80 was co-expressed with GFP-NSP5 in Vero cells, as shown in Fig 1B, NSP5 and NS80 formed separate distinctive globular structures in cells, and the globular inclusion structures formed by the two proteins did not colocalize with each other, indicating that NS80 did not associate with NSP5. However, when pCI-neo-NS80 was co-transfected with NS38-GFP-NSP5 into Vero cells, the respective globular structures completely colocalized with each other (Fig 1B). To rule out the viral inclusion bodies formed by NS80 or GFP-NSP5 were caused by protein misfolding or the possibility of unspecific aggregations, the colocalization assays between NS80 or GFP-NSP5 and poly-ubiquitination or vimentin were performed. As shown in Fig 1C and 1D, there was no colocalization between NS80 or GFP-NSP5 and poly-ubiquitin or vimentin, which demonstrated that the viral factories formed by NS80 or GFP-NSP5 in transfected cells were not induced by misfolding proteins, such as poly-ubiquitin or vimentin. These results clearly indicated that NSP5-based platform is credible and could be used to identify regions of NS80 associated with other proteins of aquareovirus.

### NS80 aa 56 to 268 Are Required for Interaction with VP1

**Fig 2:**
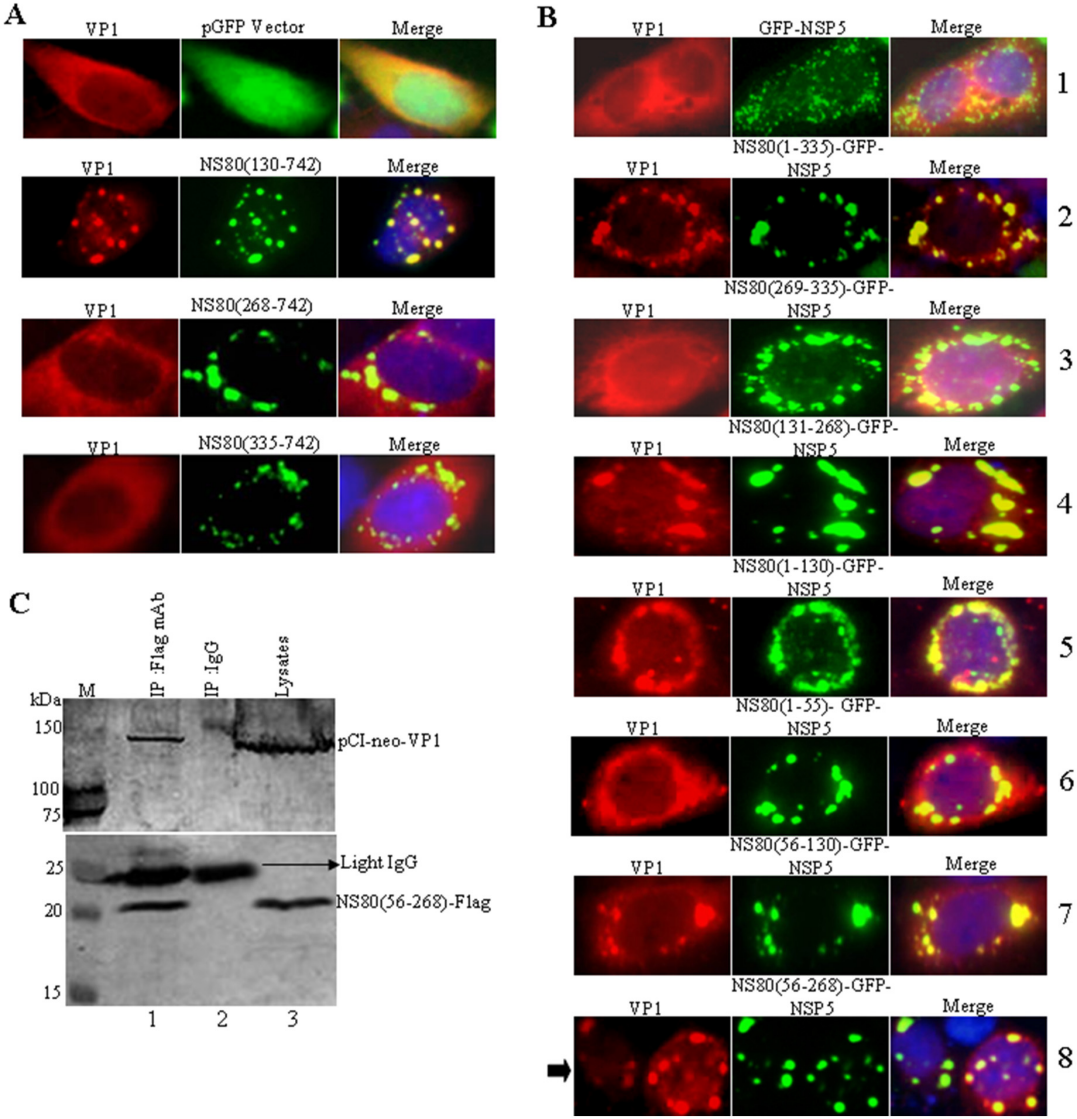
VP1 interacts with the aa 56 to 268 of NS80. (A) Vero cells were co-transfected with pCI-neo-VP1 and either pGFP vector, pCI-neo-NS80(130–742), pCI-neo-NS80(268–742), or pCI-neo-NS80(335–742). At 24 h post-transfection, cells were fixed and stained with NS80 pAbs and VP1 mAbs, respectively. And then it followed by Alexa 568-conjugated donkey anti-mouse IgG (red) and Alexa 488-conjugated donkey anti-rabbit IgG (green). Cell nuclei (blue) were stained with DAPI. (B) Vero cells were co-transfected with pCI-neo-VP1 and either GFP-NSP5 (row 1), NS80(1–335)-GFP-NSP5 (row 2), NS80(269–335)-GFP-NSP5 (row 3), NS80(131–268)-GFP-NSP5 (row 4), NS80(1–130)-GFP-NSP5 (row 5), NS80(1–55)-GFP-NSP5 (row 6), NS80(56–130)-GFP-NSP5 (row 7), or NS80(56–268)-GFP-NSP5 (row 8). At 24 h post-transfection, cells were fixed and stained with VP1 mAbs, and then followed by Alexa 568-conjugated donkey anti-mouse IgG (red). Cell nuclei (blue) were stained with DAPI. The arrow point to the region of NS80 interacts with VP1. (C) HEK 293T cells were co-transfected with Flag-NS80(56–268) and pCI-neo-VP1. At 36 h post-transfection, cells were harvested and lysed, the samples were then subjected to co-IP assays using anti-Flag mAb or control IgG (lane 1, IP: Flag mAb. lane 2, IP: IgG. lane 3: lysates). Western blot was probed with the indicated Abs (upper panel: VP1 pAb. lower panel: Flag mAb).

Three dimensional structural reconstructions by cryo-electron microscopy have indicated that VP1 is an inner-capsid protein of aquareovirus and five VP1 molecules comprise a turret [7, 8]. As VP1 has been deduced to be a capping enzyme, it may be involved in the capping process of nascent RNA transcripts in primary transcription cycle during infection [42]. Recent study in our laboratory indicated that full-length NS80 could interact with VP1 during viral infection [33]. To determine which region of NS80 was associated with VP1, a series of N-terminal deletion mutants reported previously were utilized [32]. All the plasmids expressing each deletion mutant and pGFP empty vector with the plasmid expressing VP1 were co-transfected into Vero cells and then the localization of VP1 relative to viral inclusion bodies was visualized at 20 h post-transfection by immunofluorescence microscopy. As shown in Fig 2A, VP1 could be completely colocalized with NS80(130–742). However, the distributions of VP1 in cells co-transfected with pGFP empty vector, NS80(268–742) or NS80(335–742) appeared in diffused pattern, which is consistent with VP1 in solely expressed pattern (Fig 2A). These results suggested that the sequence in the N-terminal of NS80 should be necessary for interaction with VP1.

To further define the interaction region between NS80 and VP1, the truncation NS80(1–335) tagged to GFP-NSP5 was constructed, and a series of plasmids expressing more detailed shortened fragments of NS80 fused to GFP-NSP5 were also generated. Then, the pCI-neo-VP1 plasmid was co-transfected with these plasmids (see S1 Table) into Vero cells for association analysis of expressed proteins. GFP-NSP5 served as control. As shown in Fig 2B, VP1 could colocalize with NS80(1–335)-GFP-NSP5 (row 2), NS80(131–268)-GFP-NSP5 (row 4), NS80(1–130)-GFP-NSP5 (row 5), NS80(56–130)-GFP-NSP5 (row 7), or NS80(56–268)-GFP-NSP5 (row 8), but could not associate with NS80(269–335)-GFP-NSP5 (row 3), indicating that NS80 aa 269 to C-terminus are not necessary for the interaction. In addition, more detailed truncations from both NS80 terminals indicated that the NS80(1–55)-GFP-NSP5 (row 6) could not associate with VP1. No colocalization was detected with VP1 and GFP-NSP5 (row 1). These results suggested that NS80 aa 56 to 268 are necessary for the association with VP1 (arrow indicated, row 8).

To clarify the potential direct interaction between NS80(56–268) and VP1, the plasmids pCI-neo-VP1 and Flag-NS80(56–268) were co-transfected into HEK 293T cells. And co-immunoprecipitation/Western blot analysis was performed with antibodies against Flag-tag and VP1. It appeared that VP1 was efficiently co-immunoprecipitated with NS80(56–268) by anti-Flag mAb, but not by control antibody IgG (Fig 2C, compare lanes 1 to 2). Taken together, these results confirmed that VP1 could interact with the aa 56 to 268 of NS80.

### NS80 aa 1 to 268 Are Necessary for Interactions with VP4

**Fig 3:**
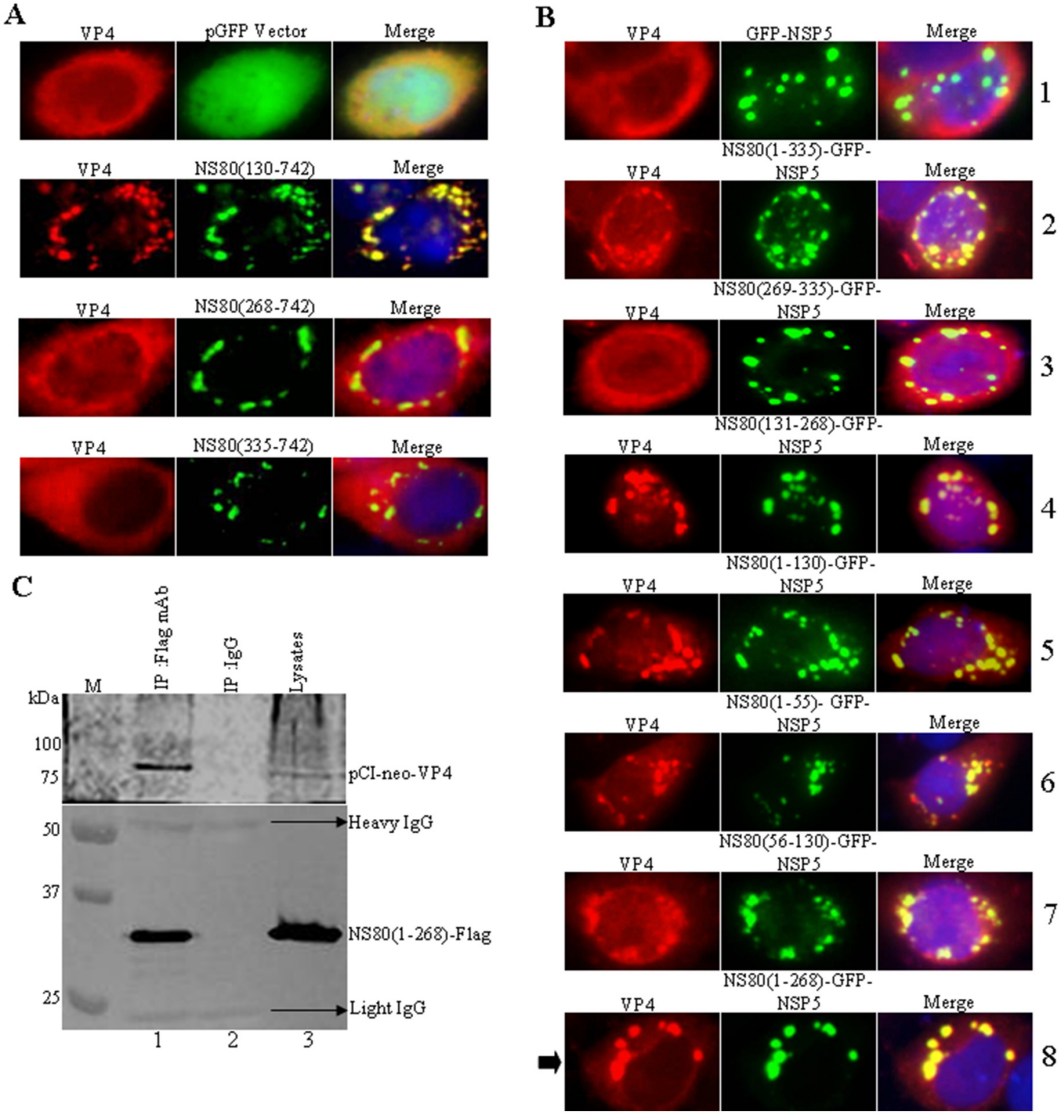
VP4 interacts with the aa 1 to 268 of NS80. (A) Vero cells were co-transfected with pCI-neo-VP4 and either pGFP vector, pCI-neo-NS80(130–742), pCI-neo-NS80(268–742), or pCI-neo-NS80(335–742). At 24 h post-transfection, cells were fixed, stained and visualized as described in Fig 2A. (B) Vero cells were co-transfected with pCI-neo-VP4 and either GFP-NSP5 (row 1), NS80(1–335)-GFP-NSP5 (row 2), NS80(269–335)-GFP-NSP5 (row 3), NS80(131–268)-GFP-NSP5 (row 4), NS80(1–130)-GFP-NSP5 (row 5), NS80(1–55)-GFP-NSP5 (row 6), NS80(56–130)-GFP-NSP5 (row 7), or NS80(1–268)-GFP-NSP5 (row 8). At 24 h post-transfection, cells were fixed, stained and visualized as described above. The arrow point to the region of NS80 interacts with VP4. (C) HEK 293T cells were co-transfected with pCI-neo-VP4 and Flag-NS80(1–268). At 36 h post-transfection, cells were lysed and subjected to co-IP assays (lane 1, IP: Flag mAb. lane 2, IP: IgG. lane 3: lysates) and Western blot analysis (upper panel: VP4 pAb. lower panel: Flag mAb) with the indicated Abs as described above.

The core component protein VP4 of aquareovirus, encoded by the viral genome segment 5, is supposed to have NTPase activity and may play a key role in viral genome transcription and replication [36]. The interaction between NS80 and VP4 has been identified previously [32, 36]. To identify the regions of NS80 that are necessary for the association with VP4, a series of NS80 deletion mutants and N-terminal fragment fusions to GFP-NSP5 were created and the ability of VP4 to associate with these proteins were examined by following plasmid co-transfection into cells. The region of NS80 required for the association with VP4 was firstly defined by using deletions from the N-terminal of NS80. As shown in Fig 3A, NS80(130–742) could recruit VP4 into its viral factories, not NS80(268–742) and NS80(335–742), suggesting that VP4 interacted with N-terminal of NS80.

To further prove the region of NS80 associated with VP4, a series of plasmids expressing fusion proteins connecting fragments of the N-terminal aa 335 of NS80 to GFP-NSP5 were co-transfected with the pCI-neo-VP1 plasmid, and then the ability of VP4 to associate with each of these fusion proteins in transfected cells were tested. In each case, protein localization was examined at 20 h post-transfection by immunofluorescence microscopy. It appeared in Fig 3B that VP4 could colocalize with NS80(1–335)-GFP-NSP5 (row 2), NS80(131–268)-GFP-NSP5 (row 4), NS80(1–130)-GFP-NSP5 (row 5), NS80(1–55)-GFP-NSP5 (row 6), NS80(56–130)-GFP-NSP5 (row 6), or NS80(1–268)-GFP-NSP5 (row 8), but not colocalize with either GFP-NSP5 (row 1) and NS80(269–335)-GFP-NSP5 (row 3). Moreover, NS80(1–55)-GFP-NSP5 could recruit VP4 into inclusion bodies, which is different from VP1 (row 6). These findings suggested that NS80 aa 1 to 268 are sufficient for the association with VP4 as arrow indicated (row 8). Further co-immunoprecipitation analysis revealed that VP4 was efficiently immunoprecipitated with the expressed protein of NS80(1–268) by anti-Flag mAb, but not by control antibody IgG (Fig 3C, compare lanes 1 to 2). These data indicated that VP4 interacted with the aa 1 to 268 of NS80.

### NS80 aa 56 to 471 Are Sufficient for Interaction with VP6

**Fig 4:**
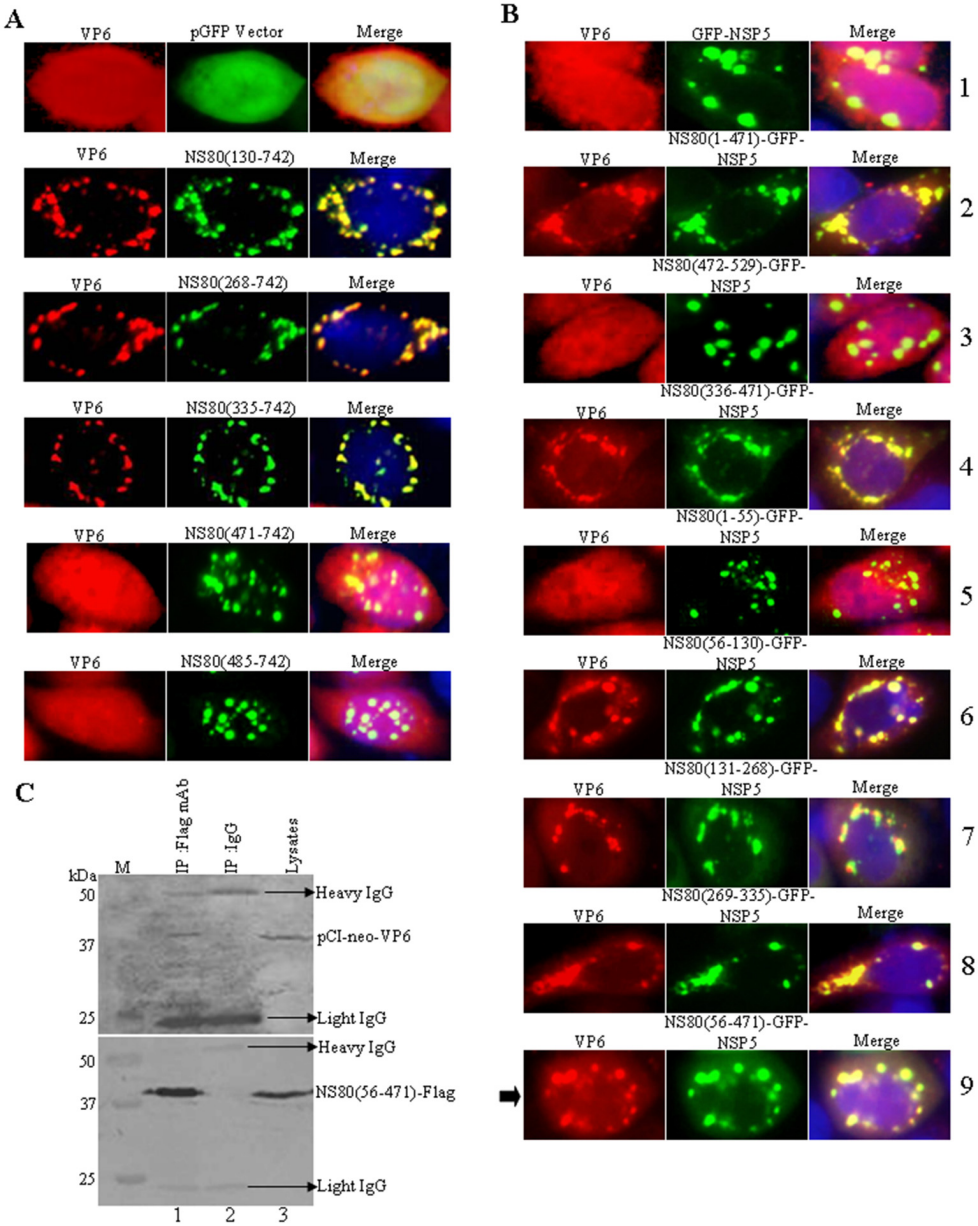
VP6 interacts with the aa 56 to 471 of NS80. (A) Vero cells were co-transfected with pCI-neo-VP6 and either pGFP vector, pCI-neo-NS80(130–742), pCI-neo-NS80(268–742), pCI-neo-NS80(335–742), pCI-neo-NS80(471–742), or pCI-neo-NS80(485–742). At 24 h post-transfection, cells were fixed, stained and visualized as described above. (B) Vero cells were co-transfected with pCI-neo-VP6 and either GFP-NSP5 (row 1), NS80(1–471)-GFP-NSP5 (row 2), NS80(472–529)-GFP-NSP5 (row 3), NS80(336–471)-EGFP-NSP5 (row 4), NS80(1–55)-EGFP-NSP5 (row 5), NS80(56–130)-GFP-NSP5 (row 6), NS80(131–268)-GFP-NSP5 (row 7), NS80(269–335)-GFP-NSP5 (row 8), NS80(56–471)-GFP-NSP5 (row 9). At 24 h post-transfection, cells were fixed, stained and visualized as described above. The arrow point to the region of NS80 interacts with VP6. (C) HEK 293T cells were co-transfected with pCI-neo-VP6 and Flag-NS80(56–471). At 36 h post-transfection, cells were lysed and subjected to co-IP assays (lane 1, IP: Flag mAb. lane 2, IP: IgG. lane 3: lysates) and Western blot analysis (upper panel: VP6 pAb. lower panel: Flag mAb) with the indicated Abs as described above.

Previous study revealed that aquareovirus core component protein VP6 could build the inner shell frame with VP3, and also VP6 has an additional role as a mediator bridging the inner core with the outer shell [8]. In this regards, VP6 may play an important role in aquareovirus replication and assembly [8]. Recent investigation in our laboratory have identified the interaction between NS80 and VP6 using yeast two-hybrid (Y2H) system and IF image [37]. To investigate which region of NS80 was associated with VP6, a series of N-terminal deletion mutants of NS80 were utilized and co-transfected with VP6 into cells. It was found that the deletion of aa 130 and 335 from the N-terminus of NS80 did not disrupt the association with VP6 (Fig 4A). Further deletions of an additional aa 150 from N-terminus, including NS80 (471–742) and NS80(485–742), resulted in the loss of an association with VP6 (Fig 4A). To identify the accurate regions of NS80 interacted with VP6, the pCI-neo-VP6 plasmid was co-transfected with a series of plasmids expressing the fragments of N-terminal of NS80 fused to GFP-NSP5 (S1 Table). IF assays were shown Fig 4B that VP6 could colocalize with NS80(1–471)-GFP-NSP5 (row 2), NS80(336–471)-GFP-NSP5 (row 4), NS80(56–130)-GFP-NSP5 (row 6), NS80(131–268)-GFP-NSP5 (row 7), NS80(269–335)-GFP-NSP5 (row 8) and NS80(56–471)-GFP-NSP5 (row 9), but could not colocalize with NS80(1–55)-GFP-NSP5 (row 5) and NS80(472–529)-GFP-NSP5 (row 3). These findings indicated that NS80 aa 56 to 471 are associated with VP6 as arrow indicated (row 9). Further co-immunoprecipitation analysis has confirmed that VP6 could be efficiently immunoprecipitated with the expressed protein of NS80(56–471) by anti-Flag mAb, but not by control antibody IgG (Fig 4C, compare lanes 1 to 2). These results demonstrated that VP6 interacted with the aa 56 to 471 of NS80.

### NS80 aa 1 to 130 Are Necessary for Interaction with NS38

**Fig 5:**
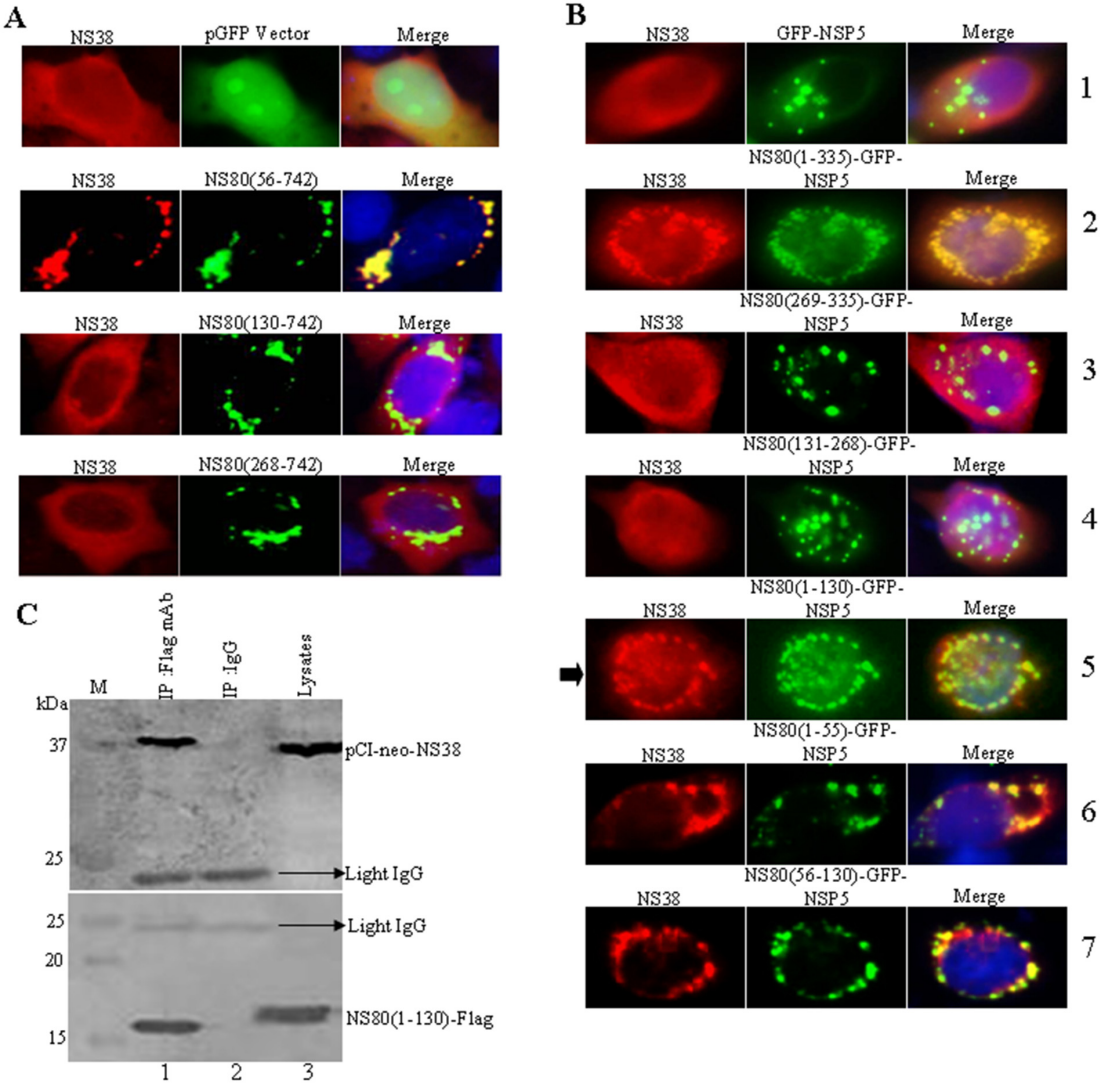
NS38 interacts with the aa 1 to 130 of NS80. (A) Vero cells were co-transfected with pCI-neo-NS38 and either pGFP vector, pCI-neo-NS80(56–742), pCI-neo-NS80(130–742) or pCI-neo-NS80(268–742). At 24 h post-transfection, cells were fixed, stained and visualized as described above. (B) Vero cells were co-transfected with pCI-neo-VP6 and either GFP-NSP5 (row 1), NS80(1–335)-GFP-NSP5 (row 2), NS80(269–335)-GFP-NSP5 (row 3), NS80(131–268)-GFP-NSP5 (row 4), NS80(1–130)-GFP-NSP5 (row 5), NS80(1–55)-GFP-NSP5 (row 6), or NS80(56–130)-GFP-NSP5 (row 7). At 24 h post-transfection, cells were fixed, stained and visualized as described above. The arrow point to the region of NS80 interacts with NS38. (C) HEK 293T cells were co-transfected with pCI-neo-NS38 and Flag-NS80(1–130). At 36 h post-transfection, cells were lysed and subjected to co-IP assays (lane 1, IP: Flag mAb. lane 2, IP: IgG. lane 3: lysates) and Western blot analysis (upper panel: VP6 pAb. lower panel: Flag mAb) with the indicated Abs as described above.

The nonstructural protein NS38 of aquareovirus, the homologous protein of MRV σNS, is supposed to have ssRNA-binding ability [3, 35]. As is reported that NS80 can interact with NS38 in infected and transfected cells [32], but the amino acid sequence of NS80 that is responsible to interact with NS38 was unknown. Similar to core proteins VP1, VP4 and VP6, we next investigated the region of NS80 that was necessary for the association with NS38 by individually co-transfecting a plasmid expressing NS38 with a panel of N-terminal deletion mutants of NS80. Immunofluorescence microscopy showed that NS38 colocalized with inclusion bodies formed by NS80(56–742) (Fig 5A). But, NS38 was diffusely distributed throughout cells when co-expressed with NS80(130–742) or NS80(268–742) (Fig 5A), which showed the same pattern as appeared in NS38 singly transfected cells.

To determine the region of NS80 associated with NS38, the pCI-neo-NS38 plasmid was co-transfected with a series of plasmids expressing the fragments of N-terminal of NS80 fused to GFP-NSP5 (S1 Table). As shown in Fig 5B that NS38 could colocalize with NS80(1–335)-GFP-NSP5 (row 2), NS80(1–130)-GFP-NSP5 (row 5), NS80(1–55)-GFP-NSP5 (row 6), or NS80(56–130)-GFP-NSP5 (row 7), but could not colocalize with NS80(131–268)-GFP-NSP5 (row 4) and NS80(269–335)-GFP-NSP5 (row 3). These results clearly indicated that NS38 could associate with the region aa 1 to 130 of NS80 as arrow indicated (row 5). Further co-immunoprecipitation analysis has revealed that NS38 was efficiently immunoprecipitated with the expressed protein of NS80(1–130) by anti-Flag mAb, but not by control antibody IgG (Fig 5C, compare lanes 1 to 2). Taken together, these results indicated that NS38 interacted with the aa 1 to 130 of NS80.

### The N-Terminal of NS80 Is Required for Viral Replication

**Fig 6:**
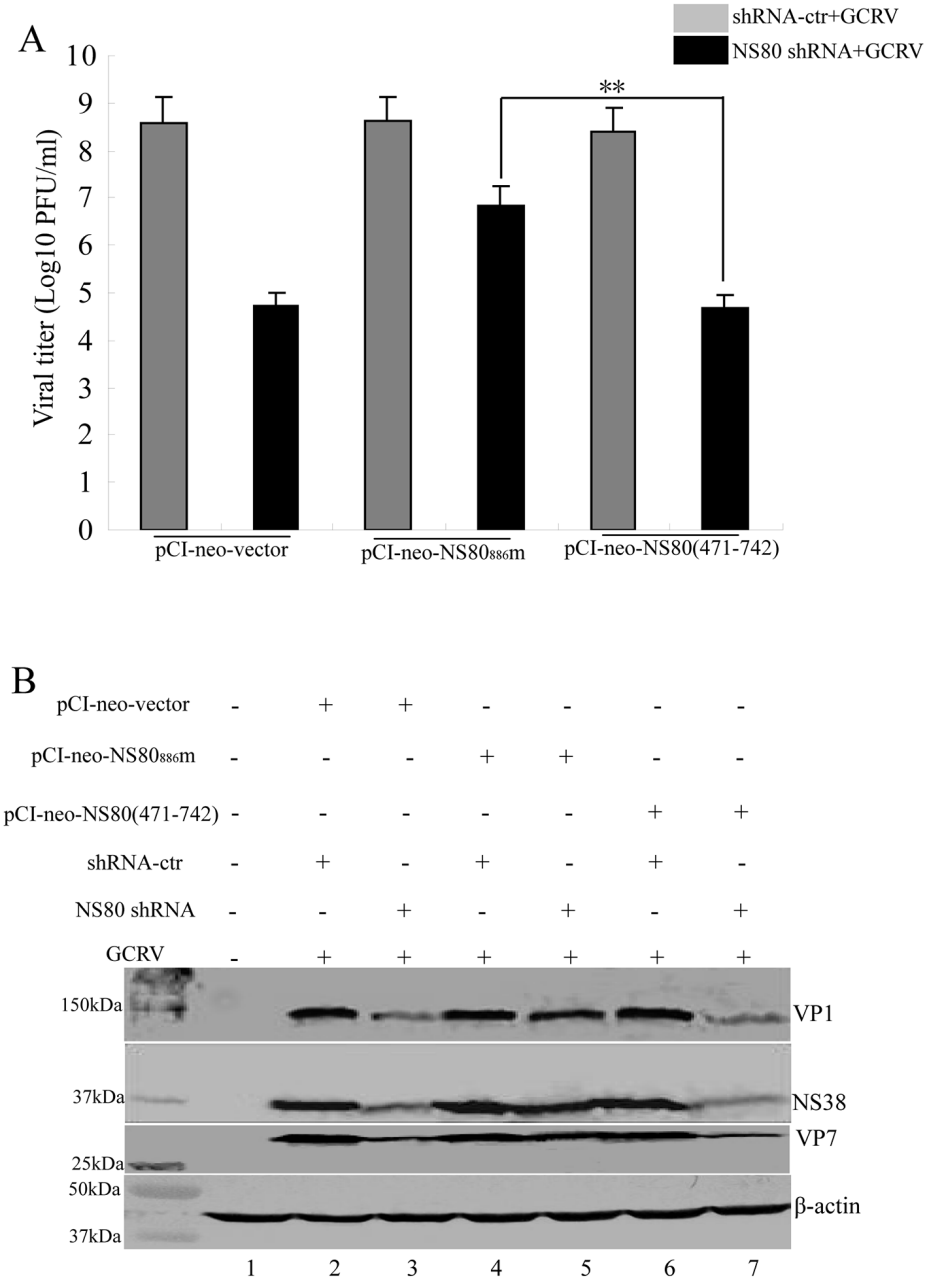
Full length NS80 complemented aquareovirus replication in NS80 shRNA_886_-based replication complementation assays. (A) Viral titers were examined by plaque assays. CIK cells were co-transfected with shRNA-control or NS80 shRNA_886_ and either with pCI-neo-vector, pCI-neo-NS80_886m_, or pCI-neo-NS80(471–742) plasmid, and then infected with GCRV at MOI of 1 at 24 h post-transfection. Cell supernatants were collected at 24 h post-infection and virus titers were tested by plaque assays. The data represent means plus standard deviations for three independent experiments. Statistical analysis was performed using Student’s t test. ** indicates P<0.01. Error bars denote standard deviations. (B) Viral proteins expression were analyzed by Western blot. CIK cells were mock-transfected, or co-transfected with shRNA-control or NS80 shRNA_886_ and either with pCI-neo-vector, pCI-neo-NS80_886m_, or pCI-neo-NS80(471–742) plasmid, and then infected with GCRV at MOI of 1 at 24 h post-transfection. CIK cells were mock transfected (lane 1), or co-transfected with shRNA-control or NS80 shRNA_886_ and either with plasmid pCI-neo-vector (lanes 2 and 3), pCI-neo-NS80_886m_ (lanes 4 and 5), or pCI-neo-NS80(471–742) (lanes 6 and 7), Cells were lysed and subjected to Western blot analysis with the indicated Abs, and β-actin was used as loading control.

Previous reports indicated that knockdown of NS80 by shRNA-2, which is named as shRNA_886_ in this study, could restrain viral replication and the C-terminal of NS80 was indispensable for viral inclusion bodies formation [32, 33]. The aforementioned results showed that the N-terminal of NS80 was required for interaction with VP1, VP4, VP6, and NS38. To determine whether the N-terminal sequences of NS80 are required for viral replication, the expression plasmid pCI-neo-NS80_886m_ containing three silent point mutations within the NS80 shRNA_886_ target sequence was constructed for trans-complementation analysis of the virus replication. The CIK Cells expressing NS80 ShRNA_886_ were transfected with the plasmids pCI-neo-vector, pCI-neo-NS80_886m_, or pCI-neo-NS80(471–742) respectively, and then infected with GCRV at MOI of 1 at 24 h post-transfection. Cell supernatants were harvested for viral titer assay. As shown in Fig 6A, the virus titer reduced more than 3.8log10 PFU in NS80 shRNA_886_ transfected cells in comparison to the cells transfected with shRNA-control. But, when pCI-neo-NS80_886m_ was co-transfected with NS80 shRNA_886_, the virus titer was rescued about 2.3log10 PFU, the detailed data is available in Data B in S1 File. Meanwhile, when pCI-neo-NS80(471–742) was co-transfected with NS80 shRNA_886_, the virus titer remained almost similar to that of cells transfected with NS80 shRNA_886_, indicating that N-terminal sequences of NS80 are critical for viral replication. To further prove whether the N-terminal sequences of NS80 influence the expression of viral proteins, the whole-cell lysates were examined by Western blot using antibodies against inner-capsid protein VP1, outer-capsid protein VP7 and nonstructural protein NS38 of GCRV. As shown in Fig 6B, when NS80 shRNA_886_ was co-transfected with empty pCI-neo vector, the expressions of VP1, NS38, and VP7 were partly inhibited (lane 3). As expected, the expressions of VP1, NS38, and VP7 could be largely restored when NS80 shRNA_886_ was co-transfected with pCI-neo-NS80_886m_ (lane 5). However, it was not able to obtain an obvious retrieval of the expressions of VP1, NS38, and VP7 while NS80 shRNA_886_ was co-transfected with pCI-neo-NS80(471–742) (lane 7). These results indicated that the complementation efficiency obtained using pCI-neo-NS80(471–742) was not equivalent to that effected with pCI-neo-NS80_886m_, which expresses full length NS80. These results also revealed that the N-terminal of NS80 has a critical role in supporting viral replication and viral proteins expression.

## Discussion

Reovirus replication occurs within viral inclusion bodies, which are specialized cytoplasmic compartments formed by viral structural and nonstructural proteins as well as cellular proteins. It has been reported that reoviruses, such as the MRV, bluetongue virus, rotavirus and phytoreovirus, could retain their viral proteins and newly synthesized viral RNAs within viral factories to promote the efficient viral replication [18, 28–30]. Recent study in our lab has shown that aquareovirus NS80 retained inner-capsid proteins VP1 to VP4, VP6 and nonstructural protein NS38 and the plus-strand RNAs within its inclusions during infection [32, 33]. In this study, it has revealed that the N-terminal of NS80 was not only required for interacting with VP1, VP4, VP6 and NS38, but also critical for viral replication in aquareovirus life cycle.

The globular inclusion bodies formed by protein has usually been supposed being involved in either native or misfolded states [43]. To prove if rotavirus NSP5-based protein interaction platform could induce misfolded protein or a false positive, the multiple colocalization assays between GFP-NS80, GFP-NSP5, NS38-GFP-NSP5 and the poly-ubiquitin or vimentin were conducted in this study. The results showed that the VIBs formed by NS80 or NSP5 have no colocalization with poly-ubiquitin or vimentin, suggesting that the globular inclusion bodies formed by fusion proteins were not misfolding, and the interactions between NS80 fragments and viral proteins examined by NSP5-based platform were specific interactions.

Previous studies in our lab indicated that NS80 is not only the primary driving force for viral inclusion formation but also able to coordinate the expression of viral structural proteins and viral replication. Earlier report from Shao *et*.*al* found that the C-terminal of NS80 was crucial for viral factories formation, and the nonstructural protein NS38 was retained within inclusion bodies by interacting with NS80 [32]. Later, Yan *et*.*al* identified that inner-capsid proteins VP1-VP4, VP6 and newly synthesized viral RNAs were retained within NS80-formed inclusions by interacting with NS80, and also demonstrated that knockdown of NS80 by shRNA not only inhibited the expression of aquareovirus structural proteins, but also inhibited viral infection [33]. Based on previous findings, using established rotavirus NSP5-based protein interaction platform, the general interaction regions between NS80 and proteins were defined in this study. It was found that NS80 aa 56–268 and aa 56–471 directly interacted with VP1, VP6, respectively. And NS80 aa 1 to 268 or aa 1–130 directly interacted with VP4 or NS38, respectively. These results indicated that the N-terminal region (aa 1–471) of NS80 was important for interacting with VP1 to VP4, VP6 and NS38. The results of proteins VP1,VP4, VP6 as being interaction with N-terminal NS80 were consistent with their homologous proteins λ2, μ2 and σ2 in MRV, which also associated with N-terminal of μNS [18], but different from another report that proteins VP1 and VP4 of aquareovirus associated with C-terminal regions of NS80 [44]. To further prove the different interaction regions of NS80 with viral proteins, co-immunoprecipitation analysis was also conducted in this study, and the results are consistent with immunofluorescence assays. The results suggest that the N-terminal of NS80 also plays an important role that is same as the C-terminal of NS80 in viral inclusion bodies formation and genome replication.

Earlier reports showed that proteins VP1, VP4, VP6 were transcriptional active core components, which were involved in aquareovirus transcription and replication [8, 36]. And NS38, the homologue of σNS in MRV, is recognized as ssRNA binding protein [3, 35, 45]. Given that proteins VP1, VP4, VP6 and NS38 are important element in viral replication and were identified bearing interactions with NS80 in the N-terminus, it is supposed that the N-terminal region of NS80 that interacted with these proteins might be required for viral replication. To prove this hypothesis, a complementation approach for functional analysis of NS80 in cells expressing NS80-specific shRNAs was used in this study. The complementing plasmid pCI-neo-NS80_886m_ contains three nucleotide substitutions in the NS80 shRNA_886_ encoding sequence, resulting in resistance to shRNA-mediated degradation. And based on identified interaction regions between NS80 and proteins VP1, VP4, VP6 and NS38, the recombinant plasmid pCI-neo-NS80(471–742) was utilized in the complementation analysis. It was found that transient expression of pCI-neo-NS80_886m_ was capable of rescuing viral replication in GCRV-infected cells when co-transfected with NS80 shRNA_886_. However, when pCI-neo-NS80(471–742) co-transfected with NS80 shRNA_886_, it was incapable of restoring viral replication, which indicated that removal of NS80 N-terminal sequences (aa 1–470) required for interacting with proteins VP1, VP4, VP6 or NS38 not only reduced the capacity of NS80 to support viral growth, but also prevented the expression of aquareovirus proteins. These results are according with previous report in MRV that the amino terminus aa 1–40 of μNS are essential for viral replication in reovirus infected cells, and mNSC was incapable of supporting viral growth in μNS silenced cells, pointing to the significance of interactions between μNS and μ2 or σNS for viral replication [18, 46, 47]. The more detailed molecular mechanism of aquareovirus NS80 played in viral replication is currently in investigation.

In conclusion, the functional regions of NS80 interacted with the inner-capsid proteins VP1, VP4, VP6 and nonstructural protein NS38 were defined in this study. The proteins VP1, VP4, VP6 and NS38 were found to interact with the N-terminal of NS80. Using complementation shRNA assay, we identified that the removal of N-terminal sequences (aa 1–470) of NS80 required for interacting with these proteins prevented viral replication. To our knowledge, this is first time to indicate N-terminal of NS80 was important in supporting aquareovirus replication. The results provided strong experimental evidence for further revealing the molecular mechanisms of NS80 interacting with viral proteins in great details as well as cellular proteins in aquareovirus live replication cycles.

## Supporting Information

S1 File The raw data for co-immunoprecipitation and viral titers analysis. The detailed data of co-immunoprecipitation assays (Fig A) and viral titer analysis results (Data B).
(RAR)

S1 Table The plasmids expressing NS80 fragments fused to EGFP-NSP5. (DOC)

S2 Table The primers for construction of plasmids expressing NS80 truncations. (DOC)
