# Supplementary material for: The N-Terminal of Aquareovirus NS80 Is Required for Interacting with Viral Proteins and Viral Replication
Source: PLoS One. 2016 Feb 12;11(2):e0148550. doi: 10.1371/journal.pone.0148550 (PMC4752286; doi:10.1371/journal.pone.0148550)
Supplement: S1 Table — (DOC) [file pone.0148550.s002.doc]

**S1 Table. The plasmids expressing NS80 fragments fused to EGFP-NSP5**

| p-NS80(1-55)-GFP-NSP5 | p-NS80(1-130)-GFP-NSP5 | p-NS80(1-268)-GFP-NSP5 |
| --- | --- | --- |
| p-NS80(1-335)-GFP-NSP5 | p-NS80(1-471)-GFP-NSP5 | p-NS80(56-130)-GFP-NSP5 |
| p-NS80(56-268)-GFP-NSP5 | p-NS80(56-471)-GFP-NSP5 | p-NS80(131-268)-GFP-NSP5 |
| p-NS80(269-335)-GFP-NSP5 | p-NS80(336-471)-GFP-NSP5 | p-NS80(472-529)-GFP-NSP5 |
