# Supplementary material for: The N-Terminal of Aquareovirus NS80 Is Required for Interacting with Viral Proteins and Viral Replication
Source: PLoS One. 2016 Feb 12;11(2):e0148550. doi: 10.1371/journal.pone.0148550 (PMC4752286; doi:10.1371/journal.pone.0148550)
Supplement: S2 Table — (DOC) [file pone.0148550.s003.doc]

**S2 Table. The primers for construction of plasmids expressing NS80 truncations**

| Construct | Primers(5’to 3’) |
| --- | --- |
| pGFP-N3-NS80(1-471) | F: CGGAATTCATGGCACGCCGCATTACTTTG |
|  | R: CGGGATCCGGCCTTGGTAGCTTGGTAG |
| pGFP-N3-NS80(1-335) | F: CGGAATTCATGGCACGCCGCATTACTTTG |
|  | R: CGGGATCCGGAGATGTCTTCGGTGGGAG |
| pGFP-N3-NS80(1-268) | F: CGGAATTCATGGCACGCCGCATTACTTTG |
|  | R: CGGGATCCGTGTGAAGGTCGACGGGGGACAAC |
| pGFP-N3-NS80(1-130) | F: CGGAATTCATGGCACGCCGCATTACTTTG |
|  | R: CGGGATCCGCGCAGAAGCACGAGTTCGC |
| pGFP-N3-NS80(1-55) | F: CGGAATTCATGGCACGCCGCATTACTTTG |
|  | R: CGGGATCCCTGGTTGAAGGTTGACGGTAG |
| pGFP-N3-NS80(56-130) | F: CGGAATTCATGATTTTCGAATTTAATG |
|  | R: CGGGATCCGCGCAGAAGCACGAGTTCGC |
| pGFP-N3-NS80(56-268) | F: CGGAATTCATGATTTTCGAATTTAATG |
|  | R: CGGAATTCATGGCACGCCGCATTACTTTG |
| pGFP-N3-NS80(56-471) | F: CGGAATTCATGATTTTCGAATTTAATG |
|  | R: CGGGATCCGGCCTTGGTAGCTTGGTAG |
| pGFP-N3-NS80(131-268) | F: CGGAATTCATGTTTGCTGACCTGGCCACTCG |
|  | R: CGGAATTCATGATTTTCGAATTTAATG |
| pGFP-N3-NS80(269-335) | F: CGGAATTCATGATCGCTGATGACGAGAC |
|  | R: CGGGATCCGGAGATGTCTTCGGTGGGAG |
| pGFP-N3-NS80(336-471) | F: CGGAATTCATGCTCCTTACCCTTCACAAC |
|  | R: CGGGATCCGGCCTTGGTAGCTTGGTAG |
| pGFP-N3-NS80(472-529) | F: CGGAATTCATGTGGCTTCGTGGCCACTTG |
|  | R: CGGGATCCCTGACGTTGAAGAGCGGTGTTG |
| pCI-neo-NS80(56-742) | F: CGGAATTCATGATTTTCGAATTTAATG |
|  | R: CATTCTAGACGCTTACAGCAGCAGGGAGGC |
